# Screening of Comprehensive Panel of Cultivated and Wild *Vigna* Species for Resistance to Pulse Beetle, *Callosobruchus chinensis* L.

**DOI:** 10.3390/biology12060781

**Published:** 2023-05-27

**Authors:** Prince Sahu, Mahendra Singh, Rakesh Pandey, Mukesh Kumar Mishra, Akhilesh Kumar Singh, Bhupendra Kumar Singh, Surendra Kumar Singh, Ashutosh Rai, Vishal Chugh, Gaurav Shukla, Saurabh Singh, Kartikey Singh, Mukul Kumar, Chandra Mohan Singh

**Affiliations:** 1 Department of Entomology, Banda University of Agriculture and Technology, Banda 210 001, India; 2 Department of Plant Protection, Banda University of Agriculture and Technology, Banda 210 001, India; 3 Department of Basic and Social Sciences, Banda University of Agriculture and Technology, Banda 210 001, India; 4 Department of Statistics and Computer Science, Banda University of Agriculture and Technology, Banda 210 001, India; 5 Department of Genetics and Plant Breeding, Banda University of Agriculture and Technology, Banda 210 001, India

**Keywords:** *Callosobruchus chinensis*, mungbean, CWR, PAL, SOD, *Vigna radiata*

## Abstract

**Simple Summary:**

The most harmful storage pest of pulses is known to be the bruchid or pulse beetle, especially in the tropics and subtropics. Chemical insecticides work well to reduce bruchid infestation; however, they have negative effects on the health of food consumers. One of the best mitigating methods among efficient, safe and sustainable strategies to lessen crop losses during storage is the development of pulse-beetle-resistant cultivars. Unfortunately, the majority of pulses lack resistance to the pulse beetle. Consequently, it is necessary to look for pulse beetle resistance in wild and exotic germplasms. Identifying potential donors for pulse beetle resistance and examining their characters in bruchid-susceptible and -resistant genotypes were the goals of the present investigation. Among the tested genotypes, two genotypes, i.e., PRR 2008-2 and PRR 2008-2-sel, were found to be highly resistant, and one accession, TCR-93, was found to be resistant to the pulse beetle. The biochemical basis of resistance modulated by the basal expression of antioxidants in highly resistant genotypes has also been studied. Currently, a quicker option for generating substantial genetic gain for desired trait improvement is molecular breeding. In the present investigation, start codon targets (SCoT) markers were used to analyse the genetic differences.

**Abstract:**

Pulses are a key source of dietary proteins in human nutrition. Despite several efforts to increase the production, various constraints, such as biotic and abiotic factors, threaten pulse production by various means. Bruchids (*Callosobruchus* spp.) are the serious issue of concern, particularly in storage conditions. Understanding host–plant resistance at morphological, biochemical and molecular levels is the best way to minimize yield losses. The 117 mungbean (*Vigna radiata* L. Wilczek) genotypes, including endemic wild relatives, were screened for resistance against *Callosobruchus chinensis*; among them, two genotypes, PRR 2008-2 and PRR 2008-2-sel, which belong to *V. umbellata* (Thumb.), were identified as highly resistant. The expression of antioxidants in susceptible and resistant genotypes revealed that the activity of phenylalanine ammonia lyase (PAL) was upregulated in the highly resistant wild *Vigna* species and lower in the cultivated susceptible genotypes, along with other biomarkers. Further, the SCoT-based genotyping revealed SCoT-30 (200 bp), SCoT-31 (1200 bp) and SCoT-32 (300 bp) as unique amplicons, which might be useful for developing the novel ricebean-based SCAR markers to accelerate the molecular breeding programme.

## 1. Introduction

Legumes play an important role in human diet; therefore, they become the major contributors towards food and nutrition security. India is the country possessing the greatest advantage of growing more than a dozen pulse crops in comparison to other countries of the world. Among them, mungbean (*V. radiata* (L.) R. Wilczek) is one of the most important pulse crops belonging to family Fabaceae [1]. It is an excellent source of protein (25–28%), and other micronutrients [2,3]. It is used as food, feed and fodder. It also improves soil health by fixing the atmospheric nitrogen into the soil through symbiosis with *Rhizobium* [4,5], which enhances the yield of the subsequent crop. Despite being of economic importance, the productivity is still low due to erratic climatic conditions [6,7,8,9,10] and various biotic stresses [11,12,13,14,15]. Insect pests are the major production constraints to pulses; among them, the pulse beetle poses a serious threat to stored grains. It belongs to family Bruchidae of order Coleoptera, which causes severe economic losses. It causes significant losses in volume and consistency of stored legumes in tropical and sub-tropical regions [16]. Generally, pulse beetle infestation starts in the field, where the adult female oviposits the eggs on pods, which leads to primary infestation. Grubs penetrate into pods and remain concealed within the seeds as hidden infestation [17]. The infested seeds carry over the bruchid population to storage, leading to secondary infestation, which causes considerable damage [18]. Further, 40% seed damage has been reported in legumes by pulse beetles [19,20,21], but it can be up to 100% under heavy infestation. The ability to assess the potential of these characteristics and use them strategically in plant breeding campaigns is made possible by understanding the molecular mechanisms behind insect resistance. Biochemical pathways are capable of adversely affecting an insect’s biological functions, growth and development, as well as the impact of an attack. In order to confer resistance to bruchids, biochemical components, such as phenols, tannins, trypsin inhibitors, amylase inhibitors and antioxidants, are crucial [22,23,24]. Several studies have been performed by earlier workers on the aspect of managing the pulse beetle [25,26]. The development and deployment of pulse-beetle-resistant mungbean cultivars is one of the best mitigation strategies to reduce the crop losses during storage. Despite this, many workers have been identified the donors for pulse beetle resistance [27]. Breeding for pulse beetle resistance requires robust donors without the possibility of linkage drag [28]. Unfortunately, most of the varieties of mungbean do not possess pulse beetle resistance. Therefore, it is necessary to determine pulse beetle resistance in wild and exotic germplasms. Wild species represent a potential reservoir of many desirable genes, especially for enhancing stress resistance [29,30,31,32,33,34,35,36,37]. These donors may be utilized in breeding programmes for developing pulse-beetle-resistant mungbean varieties. 

Molecular breeding is now accelerated in mungbean after the decoding of the whole genome sequence [38]. It is now a quicker alternative for achieving high genetic gain for desired trait improvement. Various markers have been developed and utilized by earlier workers. Cross-species molecular markers have also been used by several researchers to dissect genetic and genomic variations, indicating the possibility of its utilization [39,40,41]. Development of species-specific SCAR markers may be one of the strategies to harness the potential of the wild donors in breeding programmes. Keeping the above facts under consideration, the present study was undertaken with the objective to identify the potential donors for pulse beetle resistance, expression of antioxidant enzymes and DNA fingerprinting of contrasting genotypes in bruchid-susceptible and-resistant genotypes for effective utilization in breeding programmes. 

## 2. Materials and Methods

### 2.1. Plant Material and Insect Culturing

A set of 117 *Vigna* genotypes, comprised of 101 cultivated and 16 wild genotypes, were evaluated for pulse beetle (*C. chinensis*) response (Table 1). These genotypes belong to seven different species, such as *V. radiata* (L.) R. Wilczek, *V. sublobata* (Roxb.) Verdc., *V. sylvistris* (Lukoki, Marechal and Otoul), *V. stipulacea* (Lam.) Kuntz, *Vigna glabrescens* (Maréchal et al.), *V. mungo* L. Hepper, and *V. umbellata* (Thunb.) Ohwi and H. Ohashi. The insect culture was maintained on 100 g disinfected mungbean seeds (sterilized at 60 ± 5 °C for 8 h in order to eliminate both apparent and hidden infestation of insects and mites, if any) and kept in 500 mL conical flask placed in insect growth chamber at 27 ± 2 °C temperature and 65 ± 5% relative humidity. The adults of bruchids were sexed by using key characters [42]. 

### 2.2. Screening of Test Genotypes

The experiment was performed in completely randomized design (CRD) with three replications. Five pairs of freshly emerged adults of pulse beetle per replication were used for further studies. The no-choice test was adopted for screening of test genotypes [43] with minor modifications. Briefly, after 48 h of insects being released, they were removed from the culture tubes and kept under observation in insect growth chamber under ideal conditions until the final observation recorded. The genotypes were examined on daily basis to record the observations on number of eggs laid (NEL), percent adult emergence (PAE), mean development period (MDP), growth index (GI) and percent seed weight loss (PSWL) at 30, 60 and 90 days after insect release (DAIR). The parameters were calculated as follows:

#### 2.2.1. Percent Adult Emergence

Percent adult emergence was calculated by using following formula [44]:

$$Percent\;adult\;emergence\left(S\right)=\frac{Number\;of\;adults\;emerged}{Number\;of\;eggs\;laid}\;\times \;100$$

#### 2.2.2. Mean Development Period

Mean development period is the time taken for 50% of adults to emerge. It was estimated by using the following formula [44]:

$$Mean\;Development\;Period\left(T\right)=\frac{D1A1\;+\;D2A2\;+\;D3A3+\cdots +DnAn}{Total\;number\;of\;adults\;emerged}$$

where D1 is the day at which adults started emerging (first day), A1 is the number of adults emerged on D1th day.

#### 2.2.3. Growth Index

Growth index was calculated by using the following formula [44]:

$$Growth\;Index=\frac{Log\;S}{T}$$

where S is the percent adult emergence, T is the mean development time (days)

#### 2.2.4. Seed Weight Loss (%)

The loss of seed weight was recorded at 30, 60 and 90 days after insect release by using the following formula:

$$Percent\;weight\;loss\;=\;\frac{Initial\;weight\;of\;grains\;-\;Final\;weight\;of\;grains}{Initial\;weight\;of\;grains}\;\times \;100$$

#### 2.2.5. Grouping of Test Genotypes for Pulse Beetle Reactions

Based on the growth index, the genotypes were grouped as highly resistant, HR (0.00), resistant, R (0–0.050), moderately resistant, MR (0.051–0.060), moderately susceptible, MS (0.061–0.070), susceptible, S (0.071–0.080) and highly susceptible (>0.081) as per standard procedure as suggested by [45]. 

### 2.3. Basal Expression Profiling Antioxidant

Six genotypes, that is, four HR wild ricebean genotypes belonging to *Vigna umbellata* (PRR2008-2, PRR2008-2-sel, TCR-93, L-24) and two HS mungbean (IPM 2-3, IPM 2-14), were used for basal antioxidant expression analysis. The fully mature seeds were used to study the enzymatic assay, such as basal expression of peroxidase (POD), superoxide dismutase (SOD), catalase (CAT), ascorbate peroxidase (APX), phenylalanine ammonia lyase (PAL), tannin, H_2_O_2_ and total phenol by methods suggested by Shannon et al. [46], Marklund and Marklund [47], Chance and Maehly [48], Nakano and Asada [49] and Biehn et al. [50], respectively. 

### 2.4. Molecular Genotyping

Two HR ricebean (PRR2008-2 and PRR2008-2-sel) and two HS mungbean (IPM 2-3 and IPM 2-14) genotypes from the present study were subjected to genomic DNA extraction using DNAeasy plant DNA extraction kit (Qiagen, India) following the manufacturer’s instructions. The DNA was quantified on micro-volume spectrophotometer (Qiaexpert, Germany). Finally, the DNA was normalized to a concentration of 100 ng/µL for PCR amplification. The PCR amplification was carried out in 20 µL reaction using 2X Dream Taq Green PCR Master Mix (Thermofisher Scientific, USA) and 10 pmol primers (Europhins, India) in a thermocycler (ABI, UK). PCR conditions were programmed at initial denaturation of 3 min at 95 °C, followed by 38 cycles of denaturation for 1 min at 94 °C, annealing for 1 min at 50–58 °C (primer specific), extension at 72 °C for 2 min and final extension of 72 °C for 10 min. The PCR products thus obtained were resolved on 1.2% agarose gel and gel pictures were taken using gel documentation system (E-box, Vilber, France). The 36 start codon targets (SCoT) markers were used for DNA polymorphism on selected HR and HS test genotypes to identify the unique amplicons for further developing the linked SCAR markers for effective utilization of HR genotypes in mungbean improvement. 

### 2.5. Statistical Analyses

The data were statistically analysed as per standard procedure to determine the different parameters. The treatment means were compared to least significant difference at *p* = 0.05 level of significance. One-tailed Pearson’s correlation coefficient (r) analysis was performed to determine the relationship between different growth parameters to growth index (GI). Principal component analysis (PCA) was completed to analyse the percentage of variability explained by the different components. The correlation coefficient analysis, heat map and grouping of genotypes were completed by using statistical package R version 4.01. The biochemical analysis was completed using mean, and Duncan’s multiple range test (DMRT) was applied for significance test using SPSS software version 26. The polymorphism of SCoT loci was determined by the ratio of number of polymorphic loci to total amplified loci. 

## 3. Results

### 3.1. Screening of Vigna Genotypes

**Figure 1:**
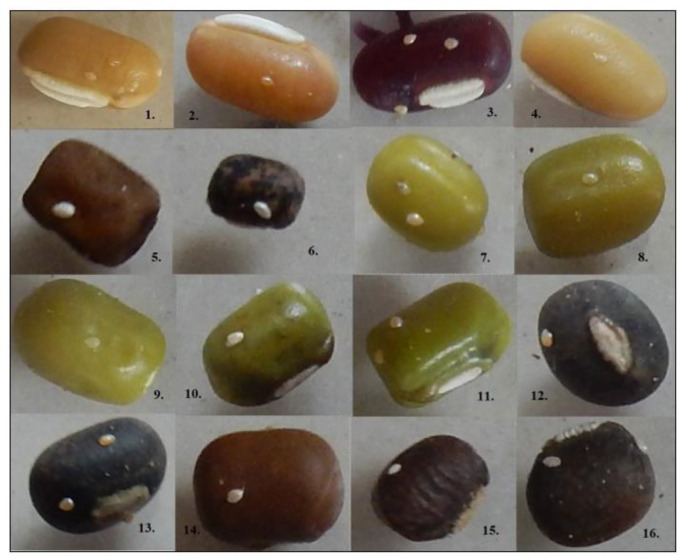
Oviposition pattern of pulse beetle on representative *Vigna* genotypes, i.e., 1. PRR 2008-2 (*V. umbellata*); 2. PRR 2008-2-sel (*V. umbellata*); 3. TCR-93 (*V. umbellata*), 4. L-24 (*V. umbellata*); 5. TCR-7 (*V. sublobata*); 6. W-17 (*V. stipulacea*); 7. IPM2-3 (*V. radiata*); 8. IPM 2-14 (*V. radiata*); 9. IPM 205-7 (*V. radiata*); 10. Shikha (*V. radiata*); 11. IMP 2K-14-9 (*V. radiata*); 12. PU-31 (*V. mungo*); 13. Uttara (*V. mungo*); 14. Banda Local 1 (*V. mungo*); 15. IPU 11-2 (*V. mungo*); 16. IPU 2-43 (*V. mungo*).

**Figure 2:**
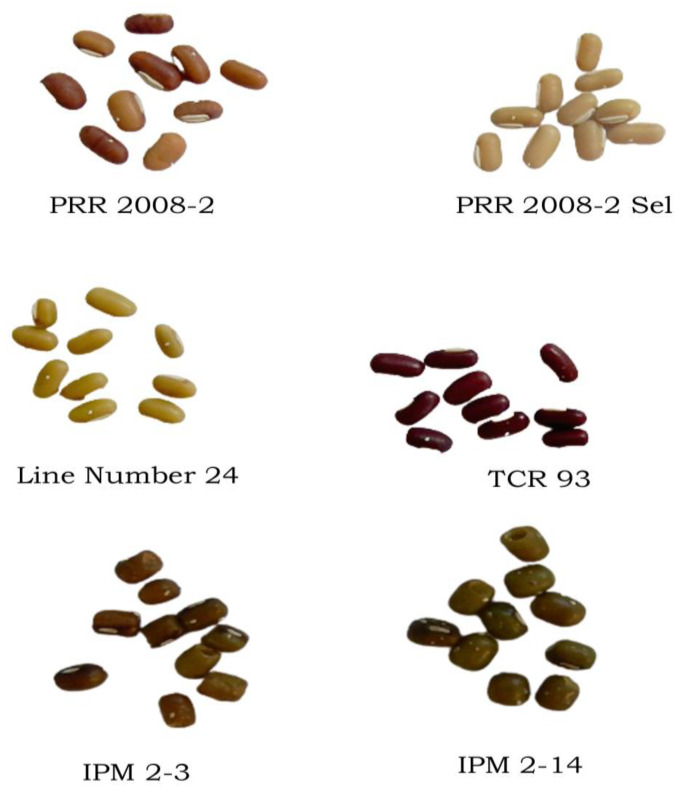
Phenotyping of representative HS and HR genotypes against bruchids; PRR 2008-2 (*V. umbellata*); PRR 2008-2-sel (*V. umbellata*); L-24 (*V. umbellata*); TCR-93 (*V. umbellata*); IPM2-3 (*V. radiata*); IPM 2-14 (*V. radiata*).

The ovipositional behaviour of pulse beetle differed significantly (*p* = 0.05) among the test genotypes (Table 1, Figure 1). The NEL ranged from 1.33 ± 0.33 (EC 496839) to 26.67 ± 0.88 (Pusa Vishal). However, no adults emerged from the two wild genotypes, i.e., PRR 2008-2 (*V. umbellata*) and PRR 2008-2-sel (*V. umbellata*). Fewer than two eggs were hatched from UPM 98-1, BMS 18-5, EC 496839, EC 520024 (*V. radiata*) and TCR 93 (*V. umbellata*), respectively. The lowest MDP (20.67 ± 0.33) was observed in BMS 18-3 (*V. radiata*), whereas the highest MDP (31.77 ± 0.50) was recorded in TCR 93 (*V. umbellata*). Likewise, the lowest growth index (GI) was recorded in PRR 2008-2 and PRR 2008-2-sel, followed by TCR 93 (*V. umbellata*), TCR-7 (*V. sublobata*) and W17 (*V. stipulacea*). The percent weight loss at 30 days after insect release (DAIR) ranged from 0.00 ± 0.00 to 42.48 ± 1.53%, with an average 22.59 ± 1.13, and it differed significantly among the genotypes. Twenty genotypes showed <10% weight loss at 30 DAIR. However, weight loss at 60 DAIR ranged from 0.00 to 54.69 ± 1.05%, with an average 27.77 ± 1.15. A total of four genotypes had <10% weight loss, i.e., PRR 2008-2, PRR 2008-2-sel, TCR 93 and L-24 (Figure 2). Similarly, percent yield loss at 90 DAIR exhibited a wider variation from 0.00 to 83.46 ± 1.01, with an average of 51.57 ± 1.44. Eight genotypes were found as resistant to pulse beetle, possessing GI ≤ 0.060. Two wild ricebean genotypes belonging to *V. umbellata,* namely PRR 2008-2 and PRR 2008-2-sel, fell under the category of HR and constituted 1.72% of the total test genotypes. One genotype, TCR 93 (also belongs to *V. umbellata),* was found as resistant to pulse beetle. Five genotypes, i.e., EC 550831, EC 520024, EC 520026 (*V. radiata*), W-17 (*V. stipulacea*) and TCR-7 (*V. sublobata*), were grouped into the MR group. Thus, a total of eight promising genotypes were found to be useful for improving the pulse beetle resistance. Further, thirty-two genotypes fell into the category of moderately susceptible, sixty-eight genotypes were under susceptible and nine genotypes were under the highly susceptible category. 

### 3.2. Correlation of Growth Index with Various Parameters

**Figure 3:**
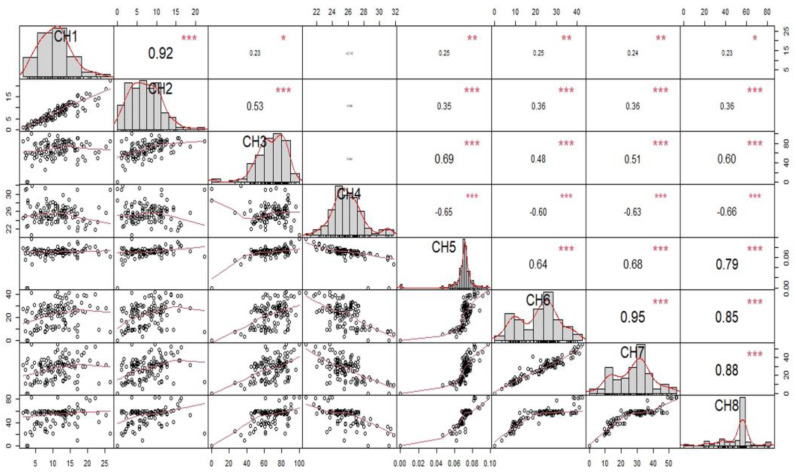
Correlation of growth index (GI) and different biological parameters. (CH1: number of eggs laid; CH2: number of adults emerged; CH3: percent adult emergence; CH4: mean development period; CH5: growth index; CH6: percent weight loss at 30 days; CH7: percent weight loss at 60 days; CH8: percent weight loss at 90 days); * = 5%, ** = 1%, ***= 0.1% level of significance.

**Figure 4:**
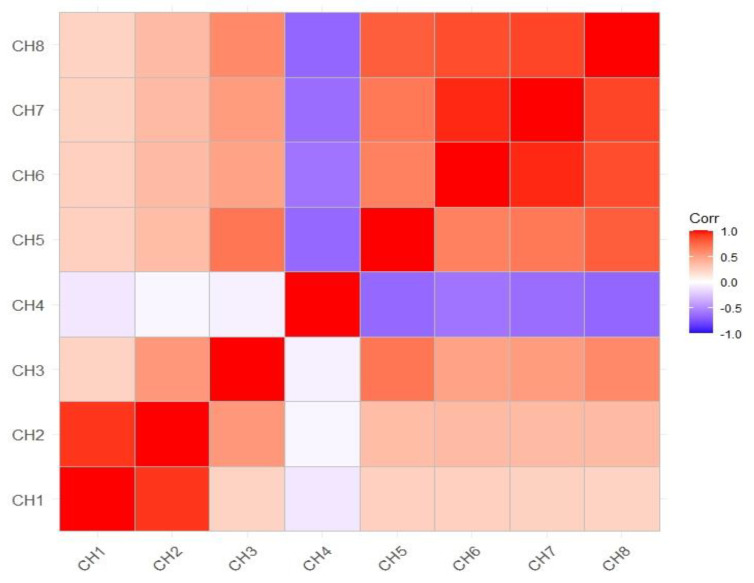
Heat map analysis of correlation coefficient exhibiting the extent of association among various parameters (CH1: number of eggs laid; CH2: number of adults emerged; CH3: percent adult emergence; CH4: mean development period; CH5: growth index; CH6: percent weight loss at 30 days; CH7: percent weight loss at 60 days; CH8: percent weight loss at 90 days).

The association of growth index with various parameters has been presented (Figure 3). A heat map of correlation analysis was also generated (Figure 4). The NEL exhibited a significant and positive correlation with number of adults emerged (r = 0.92 **), percent adult emergence (r = 0.23 **), GI (r = 0.23 *) and seed weight loss at 30 (r = 0.25 **), 60 (r = 0.25 **) and 90 DAIR (r = 0.24 **), whereas a non-significant negative correlation was found with MDP (r = −0.10). The number of adults emerged presented a significant positive correlation with percent adult emergence (r = 0.53 **), GI (r = 0.36 **), percent weight loss at 30 (r = 0.35 **), 60 (r = 0.35 **) and 90 DAIR (r = 0.36 **). The correlation of percent adult emergence was found significant and positive with GI (r = 0.60 **) and percent weight loss at 30 (r = 0.69 **), 60 (r = 048 **) and 90 DAIR (r = 0.51 **). The MDP showed a non-significant negative correlation with GI (r = −0.66**), percent weight loss at 30 (r = −0.65 **), 60 (r = −0.60 **) and 90 DAIR (r = −0.63 **). The correlation of growth index was also found to be positive and significant with NEL (r = 0.23 **), number of adults emerged (r = 0.36 **), percent adult emergence (r = 0.60 **), percent weight loss at 30 (r = 0.79 **), 60 (r = 0.85 **) and 90 DAIR (r = 0.88 **) except the MDP (r = 0.66 **). 

### 3.3. Principal Component (PC) and Cluster Analyses

**Figure 5:**
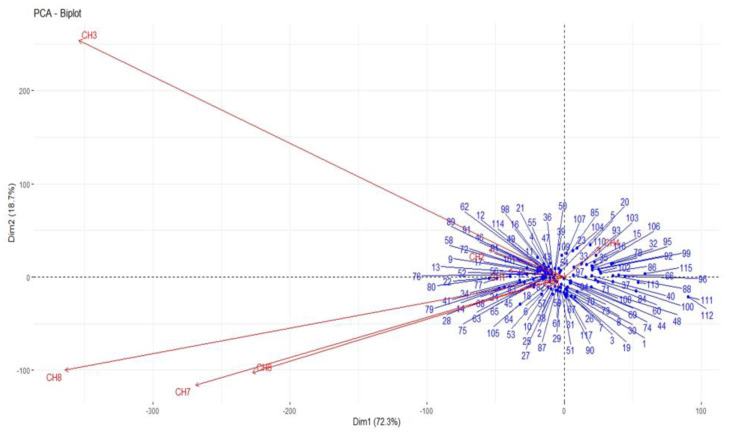
Genotype by trait biplot analysis indicating the association among various traits and plotting of genotypes based on principle components (PCs). PC1 and PC2 explained 72.3% and 18.7% variation, respectively. PRR2008-2 (G111) and PRR2008-2-sel (G112) belong to *V. umbellata* clustered together as highly resistant.

**Figure 6:**
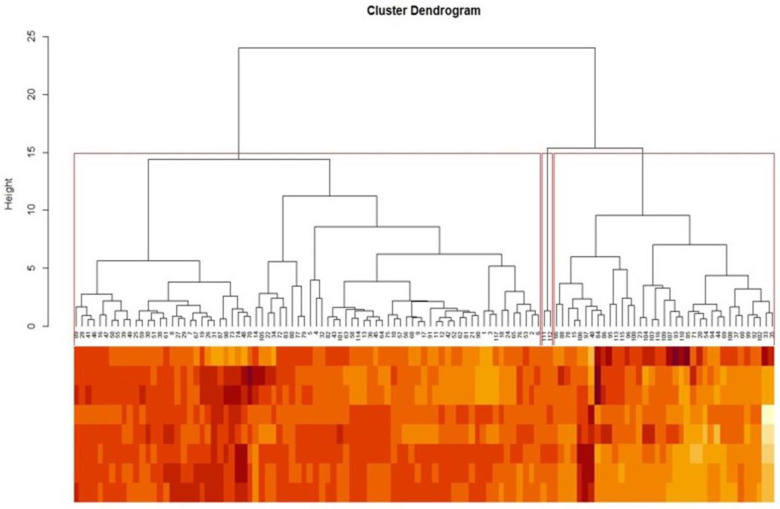
Euclidean clustering of 117 accessions of mungbean, including their wild relatives. Three main groups were formed. PRR2008-2 (G111) and PRR2008-2-sel (G112) belong to *V. umbellata* clustered together.

The four principal components (PC_1_ to PC_4_) explained about 99% of the genetic variation. Among these four PCs, two PCs with latent roots >1.00 (PC_1_ and PC_2_) accounted for about 91% of the total variation, whereas, individually, PC_1_ and PC_2_ covered 72.30% and 18.70% of variation, respectively (Figure 5). The cluster analysis of all 117 genotypes clearly distinguished them among three broad categories: HR (group I), R to MR (group II) and S to HS (group III), presented in Figure 6. The two wild genotypes, namely PRR 2008-2 (*V. umbellata*) and PRR 2008-2-sel (*V. umbellata*), grouped together, exhibiting a highly resistant response. Likewise, all the resistant to moderately resistant genotypes, such as TCR-93 (*V. umbellata*), EC550831 (*V. radiata*), EC520024 (*V. radiata*), EC520026 (*V. radiata*), TCR-7 (*V. sublobata*) and W-17 (*V. stipulacea*), grouped together in one cluster, whereas all the susceptible and highly susceptible genotypes fell into a separate cluster. 

### 3.4. Antioxidant Enzymatic Analysis

**Figure 7:**
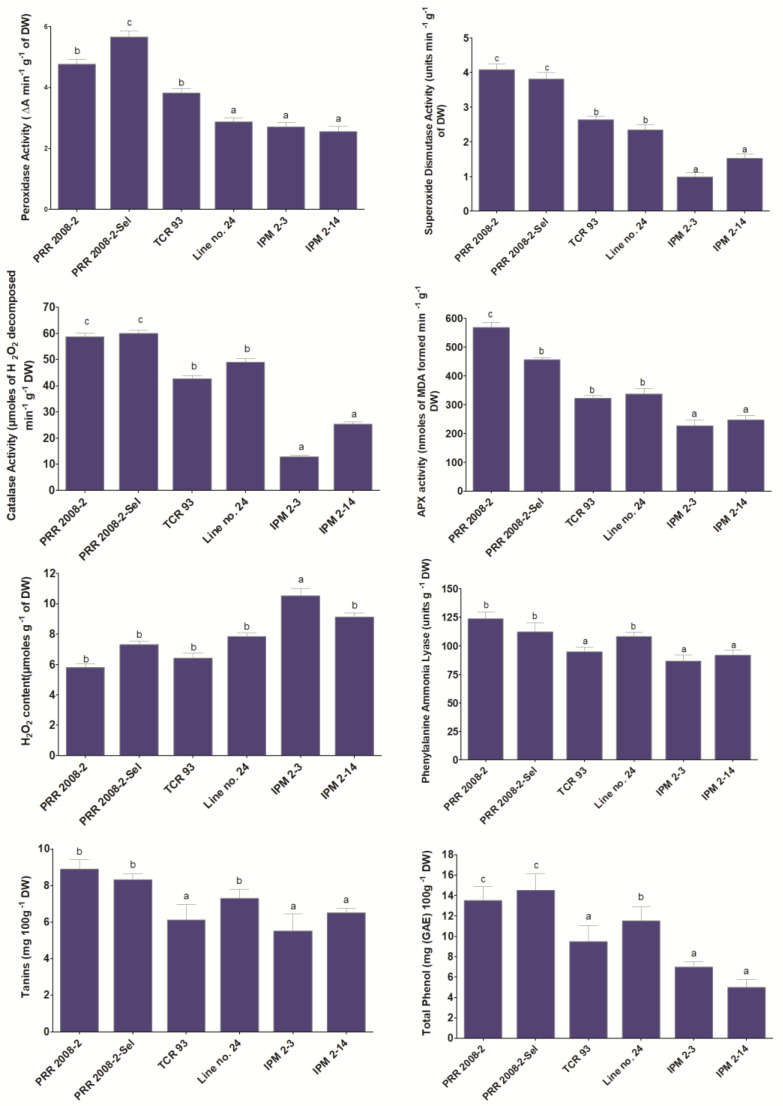
Basal expression of biomarkers in representative *Vigna* accessions. The same letter showed non-significant differences among them and vice-versa.

On the basis of screening of different *Vigna* genotypes and prevalent cultivated varieties of mungbean, four genotypes of wild *Vigna* spp., i.e., PRR 2008-2, PRR 2008-2-sel, TCR-93 and L-24, and two highly susceptible mungbean (IPM 2-3 and IPM 2-14) were selected for understanding the pattern of antioxidant expression upon oviposition of *C. chinensis* (Figure 7). The highest value of POD was recorded in PRR 2008-2-sel (5.661 ± 0.195 ΔA/min/gm of DW), followed by PRR 2008-2 (4.769 ± 0.156 ΔA/min/gm of DW). The least expression of POD was recorded in IPM 2-14 (2.555 ± 0.183 ΔA/min/gm of DW). The SOD activity ranged highest in PRR 2008-2 (4.082 ± 0.166 units/min/gm of DW) to lowest in IPM 2-3 (0.985 ± 0.132 units/min/gm of DW). The SOD activity was found about 4.14 times greater in PRR 2008-2 in comparison with IPM 2-3. The CAT activity ranged from 12.873 ± 0.503 (IPM 2-3) to 59.968 ± 1.196 (PRR 2008-2-sel) moles of H_2_O_2_ decomposed min-1 g-1 DW. PRR 2008-2-sel, a resistant genotype, has considerably increased catalase activity (4.6-fold) in comparison with the susceptible genotype. The APX activity ranged from 567.140 ± 17.964 (PRR 2008-2) to 226.109 ± 20.570 (IPM 2-3) nmoles of MDA-produced min^−1^ g^−1^ DW. The hydrogen peroxide concentration varied from 5.798 ± 0.238 (PRR 2008-2) to 10.510 ± 0.511 (IPM 2-3) moles/g of DW. Considering PRR 2008-2 regarding susceptible genotypes, the hydrogen peroxide level was roughly 1.81 times higher in IPM 2-3. The concentration of phenylalanine ammonia lyase was highest at 5.79 times in PRR 2008-2-sel, followed by PRR 2008-2 and TCR-93. The total phenol concentration was recorded highest in PRR 2008-2-sel (14.500 ± 1.640 mg (GAE) 100gm-1 DW)) and lowest in IPM 2-3 (7.000 ± 0.490 mg (GAE) 100gm-1 DW)). The tannin content was recorded highest in PRR 2008-2 (8.890 ± 0.525) and lowest in IPM 2-3 (5.510 ± 0.940). 

### 3.5. SCoT-Based Polymorphism in Selected Genotypes

**Figure 8:**
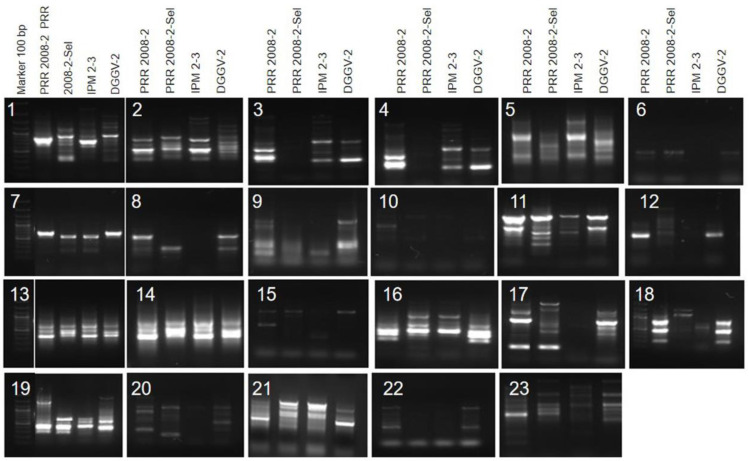
SCoT marker profiling of selected genotypes of *Vigna umbellata* (HR, lanes 1–2) and *Vigna radiata* (HS, lanes 3–4) using SCoT primers 1–23, M = Leader 100 bp.

A total of thirty-six SCoT markers were screened on a panel of four *Vigna* genotypes, including two ricebean (HR to pulse beetle) and two mungbean (HS to pulse beetle). Out of them, 23 SCoT markers produced clear and polymorphic patterns on a test panel (Figure 8). These markers were successfully amplified regarding the species, which produced 141 reliable SCoT loci, of which 83 bands were found polymorphic (Table 2). The polymorphic amplicons per primer ranged from 2 (SCoT-127) to 11 (SCoT-3), with an average of 6.13. The polymorphic bands in each primer varied from 01 (SCoT-7, SCoT-21, SCoT-24 and SCoT-35) to 09 (SCoT-3), with an average of 3.61. The polymorphisms of loci ranged from 20.00 (SCoT-35) to 10.00% (SCoT-12), with an average of 58.12%. 

A total of three SCoT markers produced species-specific fragments in ricebean and mungbean genotypes. The SCoT-30 produced about a 200 bp fragment in ricebean, whereas this loci was found absent in mungbean. Likewise, SCoT-31 produced 1200 bp and SCoT-32 produced 300 bp unique amplicon in ricebean, whereas it was absent in mungbean. These three markers might be further used for cloning and sequencing for developing SCAR markers linked to *V. umbellata* genotypes for accelerating a molecular breeding programme. 

## 4. Discussion

Development of pulse-beetle-resistant mungbean cultivars is the prime objective to minimize the losses during storage conditions. It required robust donors. In the present study, out of 117 genotypes of mungbean, including their wild relatives, the HR (PRR 2008-2 and PRR 2008-2-sel), resistant (TCR 93) and MR (EC 550831, EC 520024, EC 520026, W 17 and TCR 7) genotypes were identified. Meanwhile, moderately susceptible, susceptible and highly susceptible genotypes recorded more deposition of eggs. In the case of a broader category, i.e., susceptible genotypes and moderately susceptible genotypes, we even recorded a greater number of eggs in comparison to highly susceptible genotypes. However, adult emergence, MDP, GI and seed weight loss were lower in moderately susceptible genotypes in comparison to susceptible and highly susceptible genotypes. This may be attributed to some biochemical compounds that may be present in the seeds or seed coat, which might have inhibited the growth of bruchids. The present findings are in close proximity to the findings of previous works, where bruchids laid a maximum number of eggs on susceptible genotypes in comparison to resistant genotypes [27,51,52,53,54]. Previous works conducted by Jackai and Asante [55] indicated that oviposition preference is also affected by host range. Hence, screening of different genotypes under the present investigation was carried out with the no-choice test. Pawara et al. [56] and Revanasidda et al. [57] had also used the forcefeeding method for screening of mungbean for pulse beetle resistance. The suitability of preferential oviposition among different genotypes was determined based on emergence of adults. The seed colour of the resistant genotypes, i.e., PRR 2008-2 and PRR 2008-2-sel, and resistant genotype TCR 93 was creamish white; however, highly susceptible and susceptible genotypes were darker in seed colour, i.e., green or black. Chavan et al. [58] stated that dark-coloured cowpea seeds were more preferred for oviposition in the case of pulse beetle. In addition, the rare habit of egg laying by bruchids on unsuitable surfaces has also been observed if host conditions were not congenial [59]. The data reveal that significantly higher adult emergence was recorded in the susceptible category of genotypes in comparison to resistant genotypes. Shafique and Ahmad [60] also reported a similar trend in chickpea. The lowest MDP was recorded at 20 days in highly susceptible genotypes and the highest MDP was observed at 34 days in highly resistant genotypes. Delayed development may lead to considerable reduction in seed loss during storage conditions [45,61]. Contrary to MDP, the susceptible genotypes recorded the highest GI value of 0.099. However, the HR genotypes showed a GI value of zero. The observed results under the present investigation were similar to those of previous studies [16,62]. Seed weight loss is one of the most reliable indicators for screening of resistance genotypes [55,57]. The lowest seed weight loss was observed in the case of resistant-category genotypes, even 90 days after insect release. The present findings are also in conformity with the findings of Rawat and Srivastava [63], who observed up to 88.40% seed infestation in different genotypes of mungbean. Usha et al. [64] also observed 12.86 to 53.33% weight loss in mungbean genotypes after 90 days of insect release. The results of PCA biplot and cluster analysis supported our grouping of genotypes into different categories. The resistant against bruchid species in mungbean have earlier been reported in wild species [65,66]. Bruchid resistance has also been reported in several wild *Vigna* species, including wild black gram [67,68]. These genotypes can be utilized in breeding programmes for developing high yielding mungbean genotypes with improved pulse beetle resistance. Chen et al. [69] also used the *V. sublobata* (TC 1966) as a donor parent for gene mapping and identified 15 candidate genes on chromosome-5, including *Vradi05g03810,* for pulse beetle resistance. 

A total of eight genotypes were found under the resistant category, including HR, resistant and MR. Among eight genotypes, two wild genotypes, PRR 2008-2 and PRR2008-2-sel (*V. umbellata*), were found under highly resistant, and one wild genotype, i.e., TCR 93 (*V. umbellata*), was observed under the resistant category. Further, *V. umbellata* is crossable with *V. radiata* and produced fertile progenies [70,71,72]. Several workers [70,71,72] generated cross-combinations between *V. umbellata* × *V. radiata* for transferring the *Mungbean Yellow Mosaic Virus* resistance. Pandiyan et al. [31] transferred the pulse beetle resistance from cultivated ricebean into mungbean and identified three pulse-beetle-resistant recombinant inbred lines as RIL-158, RIL-165 and RIL-168. Mariymmal et al. [73] identified two stable QTLs, i.e., *qSD05* and *qAE08,* for pulse beetle resistance using inter-specific RILs, mapping a population of VRM (Gg) 1 × TNAU RED. This suggested that these highly resistant genotypes can be easily exploited in a breeding programme of mungbean for improving pulse beetle resistance. Apart from two highly resistant genotypes, three cultivated genotypes, EC 550831, EC 520024 and EC 520026 (*V. radiata*), and two wild genotypes, W 17 (*V. stipulacea*) and TCR 7 (*V. sublobata*), were found under MR genotypes of mungbean. Thus, these genotypes may be utilized as potential donors in a future breeding programme to develop a bruchid-resistant mungbean variety. 

Many antioxidants and detoxifying enzymes are reported to play major roles in stress tolerance [74], but the roles in bruchid resistance in wild species are not well understood. Understanding the biochemical mechanisms of plant resistance could be utilized in exploiting the trait in crop breeding. The present study investigated the expression of antioxidants and detoxifying enzymes in two *Vigna* species (*V. radiata*, *V. umbellata*) upon bruchid oviposition. The selected genotypes were subjected to antioxidant enzyme analysis. The SOD, POD, CAT and APX activities were recorded to be significantly higher in PRR 2008-2 and PRR 2008-2-sel (*V. umbellata*) as compared to other cultivated ricebean and mungbean, indicating their basal expression in the seeds. The present finding is in conformity with the findings of Mallikarjun et al. [75]. There was significant variation in the expression of H_2_O_2_ in the fresh seeds and infected seeds from *C. chinensis* of different *Vigna* genotypes both under control and *C.-chinensis*-infected seeds. The relatively lower expression of H_2_O_2_ was found in PRR 2008-2 seeds as compared to susceptible seeds. Sofo et al. [76] suggested that, at low expression, H_2_O_2_ acts as a signal molecule involved in the regulation of specific biological and physiological processes. Since stress factors provoke enhanced production of H_2_O_2_ in plants, severe damage to biomolecules can be possible due to elevated and non-metabolized cellular H_2_O_2_. Plants are endowed with H_2_O_2_-metabolizing enzymes, such as CAT, APX, etc. The PAL, tanin and phenolics were upregulated in the wild resistant genotypes, whereas lower expression was noticed in the cultivated susceptible genotypes. The present study revealed that the strong defence response activated upon the bruchid oviposition in all the wild ricebean genotypes might be a basis for the bruchid resistance in *Vigna* species. These wild accessions, i.e., PRR 2008-2, PRR 2008-2-sel, TCR-93 and L-24, may be further utilized in breeding programmes for improving the resistance towards bruchid in mungbean. 

Crop wild relatives (CWRs) also play a very important role in crop improvement [77]. Pal et al. [78] created crosses of ricebean × urdbean. Further, crossability of ricebean with urdbean indicated the possibility of developing bruchid-resistant urdbean cultivars also through introgression of desirable genes from PRR2008-2 and PRR2008-2-sel to urdbean [79]. The integration of markers technology is the most appropriate measure for shortening the breeding cycle and helps in precise and easy introduction of resistant genes into a cultivated background. The development of species-specific SCAR markers can boost the utilization of alien genes from wild relatives to cultivars for improvement. These SCAR markers will also help in identification of true interspecific hybrids. Three SCoT loci were found to be tightly linked to wild ricebean genotypes and can be used for further developing the markers for accelerating markers-assisted breeding to select the individuals possessing a ricebean genomic segment. Feng et al. [80] developed SCoT-based SCAR markers to authenticate the species of the *Physalis* species. Liu et al. [81] developed a linked SCAR marker to powdery mildew resistance in wheat; Dhole and Reddy [82] developed a marker linked to *MYMV* resistance, indicating the potential of SCAR markers in a breeding programme. 

## 5. Conclusions

Out of 117 genotypes of *Vigna*, two genotypes, PRR 2008-2 and PRR 2008-2-sel (*V. umbellata*), were identified as HR, whereas one genotype, TCR 93 (*V. umbellata*), was found as resistant to pulse beetle. These genotypes may be utilized as potential donors for development of mungbean varieties with improved resistance to pulse beetle. The basal expression of antioxidants (SOD, POD, CAT, APX, tannin and phenol) in the seeds of HR genotypes modulated the biochemical basis of resistance. The markers, SCoT-30 (200 bp), ScoT-31 (1200 bp) and ScoT-32 (300 bp), produced unique amplicons that will help in developing novel *V.-umbellata*-based molecular markers to tag the individuals possessing ricebean genomic content in the mungbean background with improved bruchid resistance. 

## Figures and Tables

**Table 1:** Screening studies of different genotypes of mungbean and their wild relatives on various parameters.

GN	Genotypes	Species	Number of Eggs Laid	No. Adults Emerged	Adult Emergence (%)	Mean Development Period (days)	Growth Index	Seed Weight Loss (%) (30 DAIR)	Seed Weight Loss (%) (60 DAIR)	Seed Weight Loss (%) (90 DAIR)
G1	PDM 139	*V. radiata*	19.33 ± 0.67	8.67 ± 0.33	45.00 ± 2.89	22.00 ± 0.58	0.075 ± 0.002	24.75 ± 1.26	29.91 ± 0.94	56.20 ± 1.86
G2	PDM 04-123	*V. radiata*	12.00 ± 1.16	7.00 ± 0.58	58.49 ± 0.83	25.33 ± 0.33	0.070 ± 0.001	37.77 ± 1.07	39.79 ± 2.22	56.79 ± 1.75
G3	PDM 281	*V. radiata*	18.67 ± 0.67	9.67 ± 0.33	51.85 ± 1.85	23.93 ± 1.10	0.072 ± 0.003	25.23 ± 0.62	30.21 ± 1.70	58.92 ± 1.42
G4	PDM 54	*V. radiata*	25.33 ± 0.88	18.67 ± 0.67	73.69 ± 0.89	25.67 ± 0.33	0.073 ± 0.001	28.39 ± 0.61	31.40 ± 1.73	52.39 ± 1.19
G5	Pusa Vishal	*V. radiata*	26.67 ± 0.88	22.33 ± 1.20	83.63 ± 1.82	31.00 ± 0.76	0.062 ± 0.001	12.49 ± 0.36	19.02 ± 0.49	24.50 ± 0.71
G6	PDM 262	*V. radiata*	14.00 ± 1.16	9.33 ± 0.88	66.57 ± 1.29	24.67 ± 0.67	0.074 ± 0.002	36.92 ± 1.36	39.42 ± 0.44	54.77 ± 1.27
G7	PDM 288	*V. radiata*	9.67 ± 0.88	4.67 ± 0.33	48.48 ± 1.52	23.33 ± 0.88	0.072 ± 0.003	27.04 ± 0.82	30.26 ± 0.65	57.44 ± 1.72
G8	PDM 178	*V. radiata*	12.67 ± 0.33	8.00 ± 0.01	63.25 ± 1.71	24.67 ± 0.33	0.073 ± 0.001	27.38 ± 2.06	32.52 ± 1.55	52.99 ± 1.35
G9	PDM 191	*V. radiata*	14.33 ± 0.88	12.00 ± 0.58	83.86 ± 1.34	26.93 ± 0.93	0.071 ± 0.003	32.09 ± 1.73	34.02 ± 1.44	58.09 ± 2.08
G10	IPM 2-14	*V. radiata*	16.33 ± 0.88	11.33 ± 0.67	69.45 ± 2.78	24.93 ± 0.52	0.074 ± 0.002	28.84 ± 1.29	34.61 ± 2.02	56.37 ± 1.39
G11	IPM 06-5	*V. radiata*	13.67 ± 0.88	11.33 ± 0.88	82.86 ± 2.35	26.83 ± 0.83	0.072 ± 0.002	25.08 ± 1.56	31.73 ± 1.07	54.10 ± 0.96
G12	IPM 409-4	*V. radiata*	13.33 ± 0.88	11.00 ± 0.58	82.65 ± 1.38	26.33 ± 0.88	0.073 ± 0.003	27.35 ± 1.18	31.49 ± 0.88	55.52 ± 2.32
G13	IPM 312-43K	*V. radiata*	11.33 ± 0.67	8.67 ± 0.33	76.67 ± 1.67	26.93 ± 1.10	0.070 ± 0.003	26.70 ± 1.70	30.98 ± 0.65	56.76 ± 1.30
G14	Selection 18-5	*V. radiata*	19.67 ± 0.33	16.33 ± 0.67	82.98 ± 2.02	23.00 ± 0.58	0.083 ± 0.002	38.91 ± 1.46	51.15 ± 1.18	81.51 ± 0.91
G15	IPM 2K-14-5	*V. radiata*	5.33 ± 0.67	3.67 ± 0.33	69.45 ± 2.78	27.07 ± 1.07	0.068 ± 0.003	8.94 ± 1.86	15.47 ± 1.39	40.95 ± 1.04
G16	Selection 18-2	*V. radiata*	9.00 ± 0.58	8.67 ± 0.67	96.30 ± 3.70	27.13 ± 1.13	0.073 ± 0.003	22.41 ± 0.61	26.47 ± 1.70	58.77 ± 2.81
G17	IPM 2-23	*V. radiata*	15.33 ± 0.33	12.67 ± 0.33	82.64 ± 2.05	26.59 ± 0.50	0.072 ± 0.001	33.37 ± 1.73	36.05 ± 2.25	55.88 ± 0.81
G18	IPM 03-1	*V. radiata*	19.00 ± 1.16	13.67 ± 0.33	72.27 ± 2.92	24.33 ± 0.67	0.077 ± 0.003	39.25 ± 0.65	45.44 ± 0.75	59.66 ± 1.15
G19	IPM 03-3	*V. radiata*	9.33 ± 0.88	5.33 ± 0.33	57.54 ± 2.50	24.33 ± 0.33	0.072 ± 0.001	25.75 ± 1.61	32.65 ± 1.52	55.06 ± 2.23
G20	IPM 2-17	*V. radiata*	11.33 ± 0.67	8.33 ± 0.67	73.33 ± 1.67	26.57 ± 1.11	0.070 ± 0.003	21.29 ± 1.29	24.58 ± 1.07	55.05 ± 0.40
G21	IPM 02-3	*V. radiata*	15.33 ± 0.67	12.67 ± 0.33	82.74 ± 1.49	26.00 ± 0.01	0.073 ± 0.001	22.30 ± 0.84	29.62 ± 0.90	54.04 ± 1.50
G22	IPM 02-3-2	*V. radiata*	17.00 ± 0.58	15.33 ± 0.33	90.40 ± 3.54	21.67 ± 0.88	0.091 ± 0.004	38.55 ± 0.86	46.00 ± 1.47	71.47 ± 0.50
G23	IPM 2-19	*V. radiata*	14.67 ± 0.67	12.67 ± 0.33	86.61 ± 3.38	28.67 ± 0.33	0.068 ± 0.001	12.62 ± 0.50	19.63 ± 0.79	37.31 ± 1.51
G24	IPM 5-2-8	*V. radiata*	18.33 ± 0.88	14.33 ± 0.33	78.38 ± 2.14	26.47 ± 0.87	0.072 ± 0.003	32.65 ± 1.50	42.39 ± 2.19	60.85 ± 1.59
G25	CO-4	*V. radiata*	7.33 ± 0.67	4.67 ± 0.33	63.89 ± 1.39	24.57 ± 0.30	0.074 ± 0.001	32.52 ± 0.94	37.38 ± 1.91	59.28 ± 3.62
G26	IPM 05-3-22	*V. radiata*	7.67 ± 0.88	4.00 ± 0.58	51.85 ± 1.85	25.22 ± 0.78	0.068 ± 0.002	27.16 ± 0.19	31.62 ± 0.30	59.32 ± 3.62
G27	IPM 306-6	*V. radiata*	9.33 ± 0.33	6.00 ± 0.01	64.45 ± 2.22	25.00 ± 0.58	0.072 ± 0.002	30.66 ± 3.13	35.10 ± 1.82	56.66 ± 2.53
G28	CO-5	*V. radiata*	4.67 ± 0.67	3.67 ± 0.67	77.78 ± 2.78	24.67 ± 0.33	0.077 ± 0.001	30.88 ± 1.60	34.57 ± 1.58	58.02 ± 1.21
G29	CO-6	*V. radiata*	11.67 ± 0.33	6.67 ± 0.33	57.07 ± 1.26	24.67 ± 0.88	0.071 ± 0.003	30.20 ± 0.50	34.23 ± 1.54	57.58 ± 0.37
G30	IPM 2K-14-9	*V. radiata*	6.67 ± 0.88	3.67 ± 0.33	55.71 ± 2.97	23.93 ± 0.52	0.073 ± 0.001	26.04 ± 2.17	29.70 ± 1.07	57.07 ± 1.16
G31	COGG-912	*V. radiata*	8.67 ± 0.67	4.67 ± 0.67	53.33 ± 3.33	24.50 ± 0.29	0.071 ± 0.001	28.44 ± 2.56	31.03 ± 0.20	60.03 ± 1.06
G32	JBT 46/23	*V. radiata*	23.33 ± 1.20	14.33 ± 0.67	61.47 ± 0.75	26.92 ± 0.92	0.066 ± 0.002	14.67 ± 0.53	21.67 ± 1.12	40.54 ± 1.76
G33	CO-7	*V. radiata*	13.67 ± 1.20	9.67 ± 0.33	71.47 ± 4.52	27.00 ± 0.58	0.069 ± 0.002	15.18 ± 0.88	17.14 ± 1.05	47.71 ± 2.00
G34	Selection 18-4	*V. radiata*	14.67 ± 0.67	12.33 ± 0.33	84.22 ± 1.49	22.33 ± 0.33	0.086 ± 0.001	41.22 ± 1.26	44.23 ± 0.42	66.24 ± 0.86
G35	Yellow Selection	*V. radiata*	16.33 ± 0.88	10.67 ± 0.88	65.14 ± 2.64	27.13 ± 1.13	0.067 ± 0.002	10.82 ± 0.64	11.75 ± 1.71	37.95 ± 1.12
G36	SML 832	*V. radiata*	13.00 ± 1.00	9.33 ± 0.67	71.86 ± 0.43	26.60 ± 0.60	0.070 ± 0.002	27.84 ± 2.41	30.45 ± 0.64	55.26 ± 1.54
G37	Pusa 9531	*V. radiata*	10.00 ± 0.58	5.67 ± 0.33	57.07 ± 4.98	25.00 ± 0.01	0.070 ± 0.002	12.22 ± 1.45	15.60 ± 1.63	43.54 ± 2.01
G38	Pusa 9972	*V. radiata*	6.33 ± 0.67	4.33 ± 0.67	67.62 ± 3.81	24.67 ± 0.33	0.074 ± 0.001	23.65 ± 1.09	32.02 ± 1.71	58.21 ± 1.09
G39	Pusa Bold 2	*V. radiata*	8.67 ± 0.33	6.33 ± 0.33	73.61 ± 6.94	24.33 ± 0.88	0.077 ± 0.004	23.71 ± 1.24	25.45 ± 2.32	58.91 ± 2.39
G40	Pusa 672	*V. radiata*	6.33 ± 1.20	2.67 ± 0.67	44.05 ± 9.74	26.33 ± 0.88	0.062 ± 0.004	10.56 ± 1.11	12.82 ± 0.96	34.21 ± 1.95
G41	Sona Green	*V. radiata*	6.33 ± 0.33	5.00 ± 0.01	79.36 ± 3.97	25.67 ± 0.33	0.074 ± 0.002	31.11 ± 2.92	33.88 ± 2.31	58.25 ± 1.50
G42	ML 818	*V. radiata*	14.67 ± 0.88	11.33 ± 0.67	77.31 ± 1.46	25.81 ± 0.43	0.073 ± 0.001	28.32 ± 1.79	30.97 ± 2.25	57.38 ± 1.24
G43	ML 5	*V. radiata*	10.00 ± 1.16	7.67 ± 0.88	76.67 ± 1.67	26.07 ± 1.21	0.072 ± 0.003	23.64 ± 1.68	25.89 ± 0.74	59.60 ± 1.17
G44	ML 512	*V. radiata*	11.67 ± 0.88	7.67 ± 0.33	66.07 ± 2.46	24.67 ± 1.33	0.074 ± 0.005	23.16 ± 1.95	25.95 ± 1.22	55.89 ± 2.63
G45	ML 515	*V. radiata*	12.33 ± 0.33	9.33 ± 0.33	75.64 ± 0.64	26.33 ± 0.88	0.071 ± 0.002	32.87 ± 1.61	35.22 ± 1.37	55.96 ± 1.62
G46	ML 682	*V. radiata*	6.33 ± 0.33	5.00 ± 0.01	79.36 ± 3.97	25.73 ± 0.73	0.074 ± 0.002	26.03 ± 1.43	32.75 ± 0.93	55.69 ± 0.72
G47	ML 729	*V. radiata*	7.33 ± 0.88	6.33 ± 0.88	85.98 ± 1.61	26.80 ± 1.33	0.072 ± 0.004	22.48 ± 0.49	24.76 ± 1.57	54.40 ± 1.20
G48	ML 1059	*V. radiata*	5.33 ± 0.33	3.33 ± 0.33	62.22 ± 2.22	24.67 ± 0.33	0.073 ± 0.001	21.26 ± 2.01	24.54 ± 1.42	55.59 ± 1.72
G49	ML 1256	*V. radiata*	8.67 ± 0.88	7.00 ± 0.58	81.16 ± 2.36	24.67 ± 0.33	0.077 ± 0.001	25.01 ± 1.48	28.59 ± 1.74	59.14 ± 0.46
G50	ML 1257	*V. radiata*	5.00 ± 0.58	4.00 ± 0.58	79.44 ± 2.42	25.40 ± 0.95	0.075 ± 0.003	20.17 ± 1.20	25.10 ± 1.83	56.47 ± 0.93
G51	AKM 96-4	*V. radiata*	6.00 ± 1.00	3.67 ± 0.67	60.83 ± 0.83	23.67 ± 0.33	0.075 ± 0.001	24.53 ± 1.60	32.49 ± 0.74	58.96 ± 1.49
G52	AKM 96-1	*V. radiata*	13.33 ± 1.20	10.33 ± 0.88	77.75 ± 3.20	26.05 ± 0.62	0.072 ± 0.001	27.29 ± 0.67	32.58 ± 1.40	58.88 ± 1.74
G53	AKM 96-2	*V. radiata*	13.67 ± 0.88	8.67 ± 0.67	63.49 ± 3.18	23.67 ± 0.67	0.076 ± 0.002	39.58 ± 2.54	48.61 ± 2.63	57.27 ± 1.93
G54	AKM/NP/8/9	*V. radiata*	12.00 ± 1.00	9.00 ± 1.00	74.61 ± 2.31	25.67 ± 0.33	0.073 ± 0.001	21.48 ± 1.76	24.59 ± 1.16	54.59 ± 2.27
G55	Pratiksha	*V. radiata*	6.67 ± 0.33	5.33 ± 0.33	80.16 ± 4.42	25.33 ± 0.67	0.075 ± 0.001	21.52 ± 0.73	26.32 ± 1.43	59.10 ± 1.41
G56	LGG 460	*V. radiata*	13.67 ± 0.88	11.33 ± 0.33	83.41 ± 4.15	26.33 ± 0.88	0.073 ± 0.003	30.83 ± 1.69	39.21 ± 0.28	58.87 ± 1.21
G57	TARAM 18	*V. radiata*	16.33 ± 0.33	11.00 ± 0.58	67.28 ± 2.45	25.33 ± 0.33	0.072 ± 0.002	24.19 ± 0.74	30.82 ± 1.63	59.03 ± 1.76
G58	TARAM 1	*V. radiata*	11.33 ± 0.67	9.67 ± 0.88	85.00 ± 3.47	27.27 ± 1.37	0.071 ± 0.004	30.13 ± 1.10	34.97 ± 0.25	58.09 ± 0.82
G59	TMB 37	*V. radiata*	6.67 ± 0.33	4.00 ± 0.01	60.32 ± 3.18	24.33 ± 0.33	0.073 ± 0.002	28.88 ± 1.43	37.76 ± 1.70	56.57 ± 1.32
G60	TMB 96-2	*V. radiata*	13.67 ± 0.67	6.67 ± 0.67	48.54 ± 2.39	24.67 ± 1.20	0.068 ± 0.002	11.76 ± 0.72	16.44 ± 1.05	42.82 ± 2.35
G61	PS 16	*V. radiata*	8.00 ± 0.58	5.00 ± 0.58	62.10 ± 2.76	25.00 ± 0.58	0.072 ± 0.002	22.30 ± 1.31	36.62 ± 0.78	58.83 ± 1.47
G62	K851	*V. radiata*	11.67 ± 1.45	10.00 ± 1.16	85.98 ± 1.61	25.67 ± 0.33	0.075 ± 0.001	24.82 ± 1.21	34.84 ± 1.23	55.43 ± 1.46
G63	MG 331	*V. radiata*	8.67 ± 0.67	6.67 ± 0.67	76.67 ± 1.67	27.33 ± 0.67	0.069 ± 0.002	30.46 ± 0.37	39.16 ± 0.66	57.86 ± 0.18
G64	Saptari	*V. radiata*	12.33 ± 0.88	8.67 ± 0.67	70.28 ± 1.84	26.33 ± 0.88	0.070 ± 0.003	29.57 ± 1.65	34.65 ± 0.67	57.85 ± 2.95
G65	Asha	*V. radiata*	10.67 ± 0.67	8.00 ± 0.01	75.56 ± 4.44	25.33 ± 0.67	0.074 ± 0.001	38.82 ± 1.18	46.48 ± 0.78	57.17 ± 2.08
G66	SPS-5	*V. radiata*	8.33 ± 0.88	4.00 ± 0.58	47.62 ± 2.38	23.33 ± 0.88	0.072 ± 0.003	12.12 ± 1.01	14.11 ± 0.69	24.62 ± 1.43
G67	Bhutan LM-1	*V. radiata*	10.67 ± 0.67	5.33 ± 0.33	50.00 ± 0.01	23.67 ± 0.33	0.072 ± 0.001	29.98 ± 1.02	33.39 ± 2.61	57.99 ± 2.38
G68	Bhutan LM-2	*V. radiata*	16.00 ± 1.00	12.33 ± 0.67	77.41 ± 4.64	26.33 ± 0.88	0.072 ± 0.003	29.14 ± 2.30	35.36 ± 1.57	57.85 ± 0.68
G69	SM 47	*V. radiata*	9.67 ± 1.20	5.67 ± 0.33	59.72 ± 5.01	24.33 ± 0.88	0.073 ± 0.002	19.75 ± 0.65	23.66 ± 1.29	56.97 ± 1.90
G70	SM 48	*V. radiata*	4.00 ± 0.58	2.33 ± 0.33	58.89 ± 4.84	25.00 ± 0.58	0.071 ± 0.003	24.44 ± 1.63	29.62 ± 0.97	55.37 ± 1.23
G71	PM-4	*V. radiata*	13.33 ± 0.88	8.67 ± 0.67	64.96 ± 1.71	25.17 ± 0.73	0.072 ± 0.002	18.34 ± 0.88	22.88 ± 1.82	48.65 ± 7.50
G72	BMS 18-1	*V. radiata*	17.33 ± 0.33	15.67 ± 0.33	90.52 ± 3.60	22.33 ± 0.33	0.088 ± 0.001	26.00 ± 0.40	36.83 ± 1.09	64.82 ± 5.53
G73	UPM 98-1	*V. radiata*	2.00 ± 0.01	1.00 ± 0.01	50.00 ± 0.01	25.00 ± 0.58	0.068 ± 0.002	17.55 ± 1.24	26.23 ± 0.84	59.26 ± 0.89
G74	UPM02-17	*V. radiata*	6.67 ± 0.88	4.00 ± 0.58	59.88 ± 1.55	23.67 ± 0.33	0.075 ± 0.001	16.11 ± 1.26	27.67 ± 1.50	56.49 ± 0.65
G75	UPM02-18	*V. radiata*	15.67 ± 1.45	11.67 ± 0.88	74.71 ± 1.36	24.67 ± 0.88	0.076 ± 0.003	25.25 ± 0.96	36.50 ± 0.81	61.16 ± 1.78
G76	HUM 12	*V. radiata*	12.67 ± 1.20	11.00 ± 1.00	86.97 ± 2.19	25.00 ± 0.00	0.078 ± 0.001	36.94 ± 2.63	45.39 ± 0.46	56.55 ± 1.64
G77	Selection 18-1	*V. radiata*	11.33 ± 0.88	10.67 ± 0.67	94.41 ± 2.83	24.00 ± 0.58	0.082 ± 0.001	38.11 ± 0.56	49.22 ± 0.74	83.28 ± 2.15
G78	EC 398885	*V. radiata*	3.33 ± 0.33	2.00 ± 0.01	61.11 ± 5.56	27.11 ± 0.59	0.066 ± 0.001	7.04 ± 0.25	10.16 ± 1.02	24.18 ± 1.70
G79	BMS 18-2	*V. radiata*	11.33 ± 0.67	9.67 ± 0.33	85.55 ± 2.22	23.67 ± 0.33	0.082 ± 0.001	42.48 ± 1.53	54.51 ± 1.81	82.86 ± 1.82
G80	BMS 18-3	*V. radiata*	3.67 ± 0.33	3.67 ± 0.33	100.00 ± 0.01	20.67 ± 0.33	0.097 ± 0.002	41.33 ± 0.66	54.69 ± 1.05	83.46 ± 1.01
G81	BMS 18-4	*V. radiata*	13.00 ± 1.16	10.67 ± 0.88	82.15 ± 1.34	24.67 ± 0.88	0.078 ± 0.003	25.42 ± 0.36	32.52 ± 0.53	57.98 ± 2.96
G82	LGG-544	*V. radiata*	8.67 ± 0.33	6.33 ± 0.33	73.15 ± 3.34	26.33 ± 0.33	0.071 ± 0.001	29.47 ± 2.18	38.70 ± 1.32	59.16 ± 0.98
G83	EC 398897	*V. radiata*	20.67 ± 1.20	16.00 ± 1.00	77.40 ± 1.22	23.43 ± 0.98	0.081 ± 0.004	30.20 ± 1.17	36.57 ± 0.54	78.44 ± 2.19
G84	EC 391178 (Y)	*V. radiata*	5.33 ± 1.20	2.67 ± 0.33	53.18 ± 7.05	24.67 ± 0.88	0.07 ± 0.005	12.92 ± 0.97	21.05 ± 1.33	38.86 ± 1.14
G85	EC 496841	*V. radiata*	13.67 ± 0.88	10.33 ± 0.67	75.63 ± 1.55	25.60 ± 0.40	0.073 ± 0.001	14.46 ± 0.95	20.13 ± 1.66	60.94 ± 2.21
G86	EC 520041	*V. radiata*	4.67 ± 0.33	2.67 ± 0.33	56.67 ± 3.33	25.67 ± 0.33	0.068 ± 0.001	8.60 ± 0.33	13.64 ± 0.42	37.44 ± 1.29
G87	Banda Local-1	*V. mungo*	2.67 ± 0.33	1.67 ± 0.33	61.11 ± 5.56	22.77 ± 0.77	0.078 ± 0.002	28.28 ± 1.45	38.40 ± 1.55	58.53 ± 0.27
G88	EC 426841	*V. radiata*	4.67 ± 0.33	2.33 ± 0.33	50.00 ± 5.77	23.67 ± 0.67	0.072 ± 0.001	12.41 ± 0.37	18.74 ± 0.53	29.40 ± 1.00
G89	EC 496839	*V. radiata*	1.33 ± 0.33	1.00 ± 0.01	83.33 ± 16.67	24.50 ± 0.76	0.078 ± 0.004	27.19 ± 0.92	32.03 ± 0.38	57.40 ± 0.63
G90	IPU 11-2	*V. mungo*	2.33 ± 0.33	1.33 ± 0.33	55.56 ± 5.56	22.67 ± 0.33	0.077 ± 0.001	25.30 ± 0.95	33.82 ± 1.57	59.43 ± 0.95
G91	IPU 2-43	*V. mungo*	13.00 ± 1.00	11.33 ± 1.33	86.66 ± 3.33	26.33 ± 0.88	0.074 ± 0.003	18.39 ± 0.73	40.76 ± 3.04	56.60 ± 1.39
G92	EC 520014	*V. radiata*	10.67 ± 0.88	6.67 ± 0.88	61.96 ± 3.32	26.67 ± 1.20	0.067 ± 0.002	10.47 ± 0.62	16.01 ± 1.25	39.01 ± 0.83
G93	EC 520016	*V. radiata*	10.67 ± 0.67	7.67 ± 0.33	72.22 ± 4.01	27.83 ± 0.44	0.067 ± 0.002	12.31 ± 0.99	19.09 ± 0.30	38.98 ± 1.68
G94	EC 520024(DR)	*V. radiata*	11.67 ± 0.88	7.00 ± 0.58	60.51 ± 5.80	25.33 ± 0.33	0.070 ± 0.002	12.13 ± 0.99	27.73 ± 2.31	57.44 ± 2.41
G95	EC 520024	*V. radiata*	2.00 ± 0.58	1.00 ± 0.01	61.11 ± 20.03	31.00 ± 0.58	0.056 ± 0.004	7.91 ± 0.44	10.54 ± 0.72	22.00 ± 1.19
G96	EC 520026	*V. radiata*	10.00 ± 0.58	4.33 ± 0.33	43.30 ± 1.67	28.13 ± 1.16	0.058 ± 0.002	9.19 ± 1.03	11.77 ± 1.38	21.92 ± 2.24
G97	EC 520029	*V. radiata*	6.67 ± 0.88	4.00 ± 0.58	59.88 ± 1.55	25.67 ± 0.33	0.069 ± 0.001	21.42 ± 1.09	23.07 ± 1.37	38.53 ± 1.73
G98	EC 520034	*V. radiata*	12.67 ± 0.88	11.00 ± 0.58	87.08 ± 1.94	25.50 ± 0.29	0.076 ± 0.001	21.87 ± 0.41	25.87 ± 0.47	59.49 ± 0.85
G99	EC 520034-1	*V. radiata*	9.33 ± 0.33	5.67 ± 0.33	60.74 ± 3.23	28.00 ± 1.16	0.064 ± 0.003	11.45 ± 1.56	14.34 ± 1.18	33.07 ± 0.61
G100	EC 550831	*V. radiata*	7.67 ± 0.33	2.00 ± 0.01	26.19 ± 1.19	25.67 ± 0.67	0.055 ± 0.002	7.43 ± 0.43	11.67 ± 1.19	24.84 ± 1.60
G101	TCR 80	*V. radiata*	10.33 ± 0.33	8.00 ± 0.01	77.58 ± 2.42	26.00 ± 0.58	0.073 ± 0.002	26.42 ± 0.60	31.16 ± 1.25	56.90 ± 0.35
G102	TCR 82	*V. radiata*	13.00 ± 0.58	7.33 ± 0.33	56.62 ± 3.44	27.27 ± 0.73	0.064 ± 0.002	9.47 ± 0.85	14.18 ± 0.50	35.57 ± 0.62
G103	TCR 7	*V. sublobata*	7.67 ± 0.33	5.67 ± 0.33	73.81 ± 1.19	31.40 ± 1.14	0.06 ± 0.002	8.21 ± 0.86	10.51 ± 0.77	29.35 ± 0.46
G104	TCR 64	*V. trilobata*	13.00 ± 0.01	11.33 ± 0.33	87.18 ± 2.56	29.67 ± 0.33	0.065 ± 0.001	14.14 ± 0.19	21.17 ± 0.80	34.74 ± 0.42
G105	DGGV 2	*V. radiata*	22.33 ± 0.88	13.67 ± 0.67	61.16 ± 1.05	21.87 ± 0.94	0.082 ± 0.004	40.41 ± 0.85	51.93 ± 0.35	81.50 ± 0.57
G106	TCR 64-1	*V. trilobata*	5.67 ± 0.33	3.67 ± 0.33	64.45 ± 2.22	27.67 ± 0.88	0.066 ± 0.002	9.24 ± 0.82	13.01 ± 0.10	39.76 ± 0.57
G107	TCR 254-1	*V. radiata*	8.00 ± 0.00	7.00 ± 0.01	87.50 ± 0.01	28.80 ± 0.76	0.068 ± 0.002	12.60 ± 0.58	14.99 ± 0.35	45.77 ± 0.43
G108	TCR 254-2	*V. radiata*	17.33 ± 0.33	8.00 ± 0.01	46.19 ± 0.87	23.67 ± 0.67	0.071 ± 0.002	13.69 ± 1.01	19.52 ± 0.64	49.59 ± 0.32
G109	TCR 262	*V. sublobata*	2.67 ± 0.33	2.33 ± 0.33	88.89 ± 11.11	28.00 ± 0.58	0.069 ± 0.001	16.00 ± 0.58	18.02 ± 0.22	47.29 ± 0.85
G110	TCR 20	*V. glabrescens*	8.33 ± 0.33	6.00 ± 0.01	72.22 ± 2.78	27.83 ± 0.73	0.067 ± 0.001	11.97 ± 0.91	13.77 ± 0.71	43.24 ± 0.47
G111	PRR 2008-2	*V. umbellata*	2.33 ± 0.33	0.00 ± 0.00	0.00 ± 0.00	0.000 ± 0.000	0.000 ± 0.000	0.00 ± 0.00	0.00 ± 0.00	0.00 ± 0.00
G112	PRR 2008-2 sel	*V. umbellata*	2.67 ± 0.33	0.00 ± 0.00	0.00 ± 0.00	0.000 ± 0.000	0.000 ± 0.000	0.00 ± 0.00	0.00 ± 0.00	0.00 ± 0.00
G113	TCR 93	*V. umbellata*	4.00 ± 0.01	1.33 ± 0.33	33.33 ± 8.33	31.77 ± 0.50	0.047 ± 0.003	5.26 ± 0.76	9.62 ± 0.53	19.14 ± 0.15
G114	TL 2	*V. stipulacea*	10.67 ± 0.33	9.00 ± 0.01	84.55 ± 2.73	27.33 ± 1.20	0.071 ± 0.003	25.74 ± 1.09	29.79 ± 0.93	55.61 ± 0.94
G115	L-24	*V. umbellata*	17.00 ± 0.58	6.33 ± 0.33	37.23 ± 1.05	25.50 ± 1.04	0.062 ± 0.002	3.45 ± 0.25	4.70 ± 0.21	9.27 ± 0.64
G116	W 17	*V. stipulacea*	9.00 ± 0.58	6.67 ± 0.33	74.26 ± 2.28	31.00 ± 0.58	0.059 ± 0.001	8.29 ± 0.18	13.00 ± 0.15	33.71 ± 0.70
G117	LRM 13-26	*V. stipulacea*	23.00 ± 0.00	11.33 ± 0.33	49.28 ± 1.45	23.67 ± 0.67	0.072 ± 0.001	27.91 ± 0.76	33.14 ± 1.27	56.83 ± 0.39

**Table 2:** SCoT marker statistics on test panel of *Vigna umbellata* and *Vigna radiata* genotypes.

SN	Primer Code	Sequence	Tm	Amplified Loci	Polymorphic Loci	Polymorphism (%)
1	SCoT-2	CAACAATGGCTACCACCC	56	10	07	70.00
2	SCoT-3	CAACAATGGCTACCACCG	53	11	09	81.82
3	SCoT-7	CAACAATGGCTACCACGG	53	04	01	25.00
4	SCoT-10	CAACAATGGCTACCAGCC	52	08	03	37.50
5	SCoT-12	ACGACATGGCGACCAACG	53	02	02	100.00
6	SCoT-13	ACGACATGGCGACCATCG	58	04	03	75.00
7	SCoT-14	ACGACATGGCGACCACGC	60	04	03	75.00
8	SCoT-15	ACGACATGGCGACCGCGA	60	05	03	60.00
9	SCoT-16	ACCATGGCTACCACCGAC	58	05	02	40.00
10	SCoT-17	ACCATGGCTACCACCGAG	60	08	04	50.00
11	SCoT-19	ACCATGGCTACCACCGGC	60	06	04	66.67
12	SCoT-21	ACGACATGGCGACCCACA	60	05	01	20.00
13	SCoT-22	AACCATGGCTACCACCAC	58	05	04	80.00
14	SCoT-24	CACCATGGCTACCACCAT	58	04	01	25.00
15	SCoT-25	ACCATGGCTACCACCGGG	56	05	03	60.00
16	SCoT-29	CCATGGCTACCACCGGCC	60	08	04	50.00
17	SCoT-30	CCATGGCTACCACCGGCG	63	07	06	85.71
18	SCoT-31	CCATGGCTACCACCGCCT	63	07	05	71.43
19	SCoT-32	CCATGGCTACCACCGCAC	60	06	05	83.33
20	SCoT-33	CCATGGCTACCACCGCAG	60	07	05	71.43
21	SCoT-34	ACCATGGCTACCACCGCA	58	06	02	33.33
22	SCoT-35	CATGGCTACCACCGGCCC	53	05	01	20.00
23	SCoT-36	GCAACAATGGCTACCACC	54	09	05	55.56
Average				6.13	3.61	58.12
Minimum				2.00	1.00	20.00
Maximum				11.00	9.00	100.00

## Data Availability

All the data generated in the experiments are presented in the manuscript.
